# Dr. S. G. Armour

**Published:** 1853-08

**Authors:** 


*Dr. S. G. Armour.*—This gentleman has lately obtained the prize offered by the State Society of Ohio, for the best essay on some subject connected with medical science. The subject of the prize essay is the Zymotic theory of Continued Fever. The essay, we presume, will be printed with the transactions of the society.

